# *In silico* analysis of cross reactivity among phospholipases from Hymenoptera species

**DOI:** 10.12688/f1000research.27089.2

**Published:** 2021-03-29

**Authors:** Yuliana Emiliani, Andrés Sánchez, Marlon Munera, Jorge Sánchez, Dilia Aparicio

**Affiliations:** 1 Health Faculty - GINUMED, Corporation University Rafael Nuñez, Cartagena, Colombia; 2 Group of Clinical and Experimental Allergy, University of Antioquia, Medellín, Colombia

**Keywords:** phospholipase, Hymenoptera, allergen, in silico, epitope, cross-reactivity.

## Abstract

**Background:** Phospholipases are enzymes with the capacity to hydrolyze membrane lipids and have been characterized in several allergenic sources, such as hymenoptera species. However, cross-reactivity among phospholipases allergens are little understood. The objective of this study was to determine potential antigenic regions involved in cross-reactivity among allergens of phospholipases using an *in silico* approach.

**Methods:** In total, 18 amino acids sequences belonging to phospholipase family derived from species of the order hymenoptera were retrieved from the UniProt database to perform phylogenetic analysis to determine the closest molecular relationship. Multialignment was done to identify conserved regions and matched with antigenic regions predicted by ElliPro server. 3D models were obtained from modeling by homology and were used to locate cross-reactive antigenic regions.

**Results:** Phylogenetic analysis showed that the 18 phospholipases split into four monophyletic clades (named here as A, B, C and D). Phospholipases from A clade shared an amino acid sequences’ identity of 79%. Antigenic patches predicted by Ellipro were located in highly conserved regions, suggesting that they could be involved in cross-reactivity in this group (Ves v 1, Ves a 1 and Ves m 1).

**Conclusions:** At this point, we advanced to the characterization of potential antigenic sites involved in cross-reactivity among phospholipases. Inhibition assays are needed to confirm our finding.

## Introduction

Allergic diseases have become a public health problem; the genetic background of patients (atopy) and the environmental conditions are considered the cause of the increased risk to develop allergic diseases ^1^. Exposure to allergens (typically harmless antigens in the environment) also promotes an immune response mediated by IgE. Over the last few years, species belonging to the order Hymenoptera have been characterized as potential allergenic sources. They represent a common source of sensitization, with more than 200,000 species including bees, wasps, and ants. Most of the member of this order are cosmopolitan species, but some of them have an endemic distribution with a capacity of sensitization , like the *Bombus* sp. located more frequently in central and northern Europe, whereas the yellowjacket (YJ) ( *Vespula* spp.) and honeybee (HB) ( *Apis mellifera*) are allergenic sources in North America. Other wasps such as Polistinae are found in southern Europe and America ^2– 4^.

Allergic immune response from hymenopteran allergens has been studied in detail due to a high incidence of sting reactions to these insects. Approximately, 9.2% to 28.7% of the adult population is sensitized to the venom of hymenopterans ^5^. Allergic response to Hymenoptera venom is one of the leading causes of anaphylaxis worldwide with a frequency of 27%, as compared to medications (41%) and foods (20%) ^3, 6^. Molecular, structural, and immunological characterization of hymenopteran venom allergens is advanced, in total 75 allergens from 31 different species have been explored, and since phospholipases are a family of allergens with clinical and biological relevance, some proteins belonging to this order such as hyaluronidase and antigen V are also considered relevant to sensitization to allergens from hymenoptera ^2, 7, 8^. Exposure to hymenoptera allergens is associated with bites and stings; it is considered that 56.6–94.5% of the general population have been bitten at least once in their life ^9^. 

Phospholipases (PLA) are a major component of the venom of these species, representing 75% of the total mass of the poison and has been characterized as one of the main allergens in Hymenoptera ^10^. They can be found in venoms from other arthropods such as chelicerates, in the venom of ophidians, as well as in different tissues of mammals such as pancreatic juice, synovial arthritic fluid. The superfamily includes 42 groups distributed in four types: A, B, C, and D ^11^.

Phospholipases belonging to class A split into two groups: class A1 hydrolyzes the phospholipid ester bond between the first acyl and glycerol (1 acyl-SN-glycerol phosphate), while class A2 hydrolyzes the bond between the second acyl and glycerol (2 Acyl-SN-glycerol phosphate). They are a family of enzymes with different molecular weights, PLA1 has a molecular weight of 28 KDa, while PLA2 are classified as high molecular weight cytosolic PLA2 (40–85 kDa) and low molecular weight secretory PLA2 (14–18 kDa) with the capacity to hydrolyze fatty acids that are present on the cell membrane and other types of lipophilic substances or participate in the mechanism of regulation of gene expression through the production of free fatty acids, from which cyclooxygenases synthesize prostaglandins ^12– 14^.

The structure, function, mechanisms, and cell signaling of PLA have been extensively studied; one important aspect of PLA is their capacity to induce allergic responses. Several epitopes involved in the co-sensitization of some PLA that share structural homology and identity have been studied; this suggests a potential role in cross-reactivity. However, this is little understood and studies are needed to complement what has been reported. The aim of this work was to explore cross-reactivity and antigenicity of allergenic PLA using an *in silico* approach, using bioinformatics tools, where we identified several antigenic regions that may be involved in cross-reactivity among phospholipases.

Today it is evident how the use of bioinformatics tools for science has grown; it is considered the first step to carry out experimental studies because they create a functional prediction. Understanding and predicting an individual clinical cross-reactivity to allergens is key to better management, treatment, and progression of new therapies for allergy to Hymenoptera; prediction can be performed by methods for the identification and computational mapping of specific IgE epitopes or epitopes reported in the Immune Epitope Database and Analysis Resource, which can help identify the conserved regions that may be affecting patients’ health. Various studies have carried out on this methodology for predicting food allergen epitopes ^15– 17^.

The *in silico* methodology has been used in other work to report possible cross-reactivity based on proteins in studies of structural or functional homology, through bioinformatics tools ^18^.

## Methodology

### Selection of phospholipases and alignment

The amino acid sequences of phospholipases type A (A1 and A2) from 18 Hymenoptera species were selected according to the allergenic capacity reported. The sequences were obtained from the UniProt database (see Table 1 for a list of accession numbers). All Allergens that were reported in the WHO/IUIS Allergen Nomenclature Sub-Committee with a complete sequence were used. We did not include incomplete sequences for analysis. Three sequences are not reported as allergenic but were chosen to observe the differences in identity and the structures of several phospholipases. The identity degree among phospholipases was determined using the PRALINE web server. The parameters to perform the alignment were configured to use BLOSUM62 as the exchange matrix. The interactions used were 3 with an E value of 0.001.

**Table 1.** Phospholipases Allergens used to compare sequences. The name of the allergen, source, and type of phospholipases and Uniprot code are detailed.

Allergens	Allergen sources	Phospholipase	Uniprot
Api c 1	Apis cerena (honeybee)	Phospholipase A2	V9IM80
Api m 1	Apis mellifera (honeybee)	Phospholipase A2	P00630
Bom p 1	Bombus pennslylvanicus (american bumblebee)	Phospholipase A2	Q7M4I6
Bom t 1	Bombus terrestri (buff-tailed bumblebee)	Phospholipase A2	P82971
Pol a 1	Polistes annulareis (paper wasp)	Phospholipase A1	Q9U6W0
Pol d 1	Polistes dominula (cardboard wasp)	Phospholipase A1	Q6Q252
Poly p 1	Polybia paulista (South America wasp)	Phospholipase A1	A2VBC4
Vesp c 1	Vespa crabro (european hornet)	Phospholipase A1	P0CH87
Dol m 1	Dolichovespula maculate (baldfaced hornet)	Phospholipase A1	P53357
Ves v 1	Vespula vulgaris (common wasp)	Phospholipase A1	P49369
Ves m 1	Vespula musculifrons (wasp of east yellow jacket)	Phospholipase A1	P51528
Ves s 1	Vespula squamosa (wasp of south yellow jacket)	Phospholipase A 1	P0CH86
Sol i 1	Solenopsis invicta (red imported fire ant)	Phospholipase A1	Q68KK0
Sol i 2	Solenopsis invicta (red imported fire ant)	Phospholipase like A1	P35775
Sol s 2	Solenopsis saevissima (red ant)	Phospholipase like A1	A5X2H7
Not allergen	Culex quinquefasciatus (house mosquito)	Phospholipase A2	B0WT10
Not allergen	Centruroides hentzi (scorpion)	Phospholipase A1	A0A2I9LPH1
Not allergen	Parasteatoda tepidariorum (house spider)	Phospholipase A2	A0A2L2Y6H2

### Phylogenetic analysis

The Molecular Evolutionary Genetic Analysis (MEGA) program, version X was used to obtain phylogenetic trees, using the method of maximum parsimony of the taxa with the support of Bootstrap with 1000 repetitions as a measure of reliability and robustness under the assumption of a minimum evolution. In the topology, this model uses a comparative matrix to find the similarity between the amino acids of 18 sequences to establish the evolutionary proximity between the species. The matrix was constructed with all the amino acid sequences of the phospholipases recovered from the UniProt database and reported to the WHO/IUIS. Therefore, the more positive identity values found between the sequences, the greater their relationship will be, and the closer they will be located in the tree. All empty spaces were eliminated (complete deletions). From the global comparison and the homologies, the sum of the length of the branches (SBL) will be presented, which will determine the number of nodes and their position, including the "groups" of the evolutionarily closest sequences. Phylogenetic sub-analyses were carried out in order to identify the degree of identity of the groups formed. The alignment for phylogenetic analysis was carried out using CLUSTAL W, which performs alignments. The parameters to perform the multiple alignment were configured to use gap opening penalty of 10.00 and gap extension penalty of 0.20, and the divergent cutoff delay was 30%.

### Generation of 3D models

The phospholipases with 3D structures not reported in the Protein Data Bank were obtained by modeling based on homology using the SWISS-MODEL server. Quality was evaluated by means of several tools, including the Ramachandran charts, WHATIF, the QMEAN4 index (The Qualitative Analysis of Energy Analysis) using ProSA-web and the SWISS-MODEL server. The results were expressed as a number between 0 and 1. Higher numbers indicate higher reliability and energy values (force field GROMOS96). ElliPro tools were used to predict lineal and conformational epitopes on a representative phospholipase for group. Residues with larger scores are associated with greater solvent accessibility. Only residues with a score > 0.7 were selected.

## Results

### Phospholipases found and phylogenetic results

We selected 15 sequences of allergenic phospholipases and three not allergenic to include in the analysis with 361 positions in the final dataset. The sequences were derived from several biological sources: two from bees, two bumblebee, six wasps, two hornet, three ants, and three sources not described as an allergen, mosquito, spider, and scorpion. The allergens of bees and wasps belong to group 1 and the ants to groups 1 and 2 ( Table 1).

**Figure 1.**
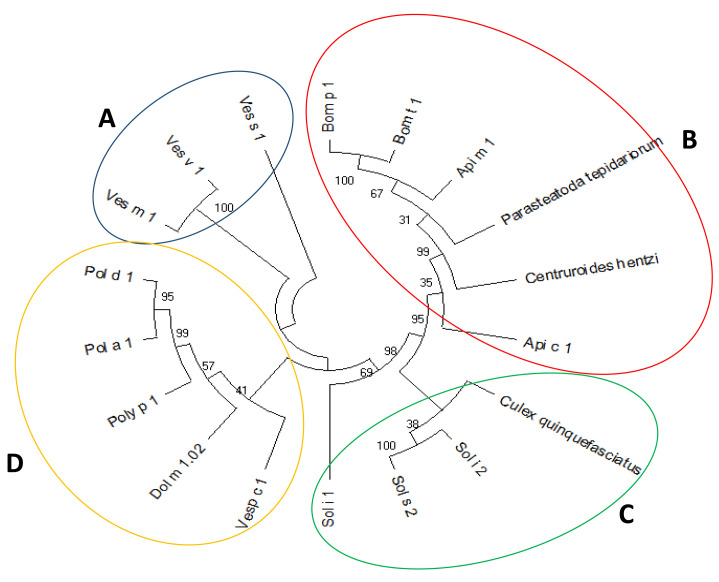
Phylogenetic tree based on the amino acid sequences of the phospholipases studied. The formation of fourth clades ( **A**– **D**) with the highest degree of identity is observed (79% for clade A). The evolutionary history was inferred using the Maximum Parsimony method. The most parsimonious tree with length equal to 1462 is shown.

The phylogenetic tree had a consistency index of 0.857256 with a retention index of 0.779682 and a composite index of 0.683688 (0.668387) for all sites and parsimony-informative sites. A closed relationship among phospholipase allergens as shown, formed four nodes with a high phylogenetic relationship among them ( Figure 1). According to the tree, group A grouped three phospholipase A1 all belonging to the *Vespula* genus (Ves v 1, Ves m 1, and Ves s 1). This group presents the greatest relationship among the groups with the closest distance between branches. Meanwhile, group B contains the highest number of phospholipases A2 phylogenetically related, including allergens of the *Bombus* and *Apis* genera (Bom p 1, Bom t 1, Api m 1, Api c 1) and two non-allergic phospholipases from *Parasteatoda tepidariorum* (Common house spider) and *Centruroides hentzi* (Hentz striped scorpion). Group C included four proteins, three of them from ants belonging to *Solenopsis* gender (Sol i 1, Sol i 2, Sol s 2) and one belonging to the mosquito *C. quinquefasciatus*. In group D we found all the wasp allergens that belong to the genus *Polistes* (Pol a 1, Pol d 1), *P. Paulista* (Poly p 1), and *D. maculate* (Dol m 1) and *V. crabro* (Vesp c 1).

### Identification of potential cross-reactive antigenic sites

**Figure 2.**
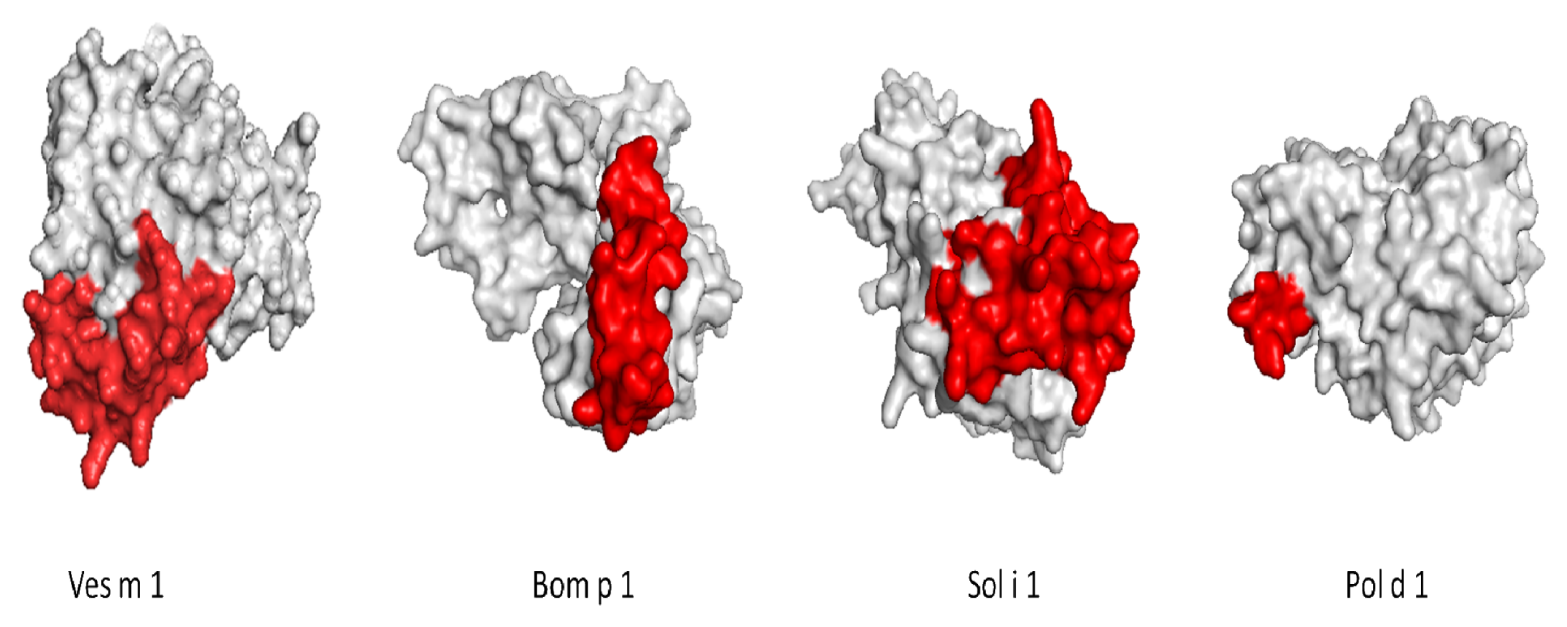
Constitutional antigenic patches with the higher score among groups. The Sol i 1 structure was obtained by homology, using the Vespid basal is sequence as a template (34.32% de identical; QMEAN -5, 45).

Multiple alignments of the phospholipases of the different groups obtained from the phylogenetic analyzes were made. We built four 3D models of the 18 phospholipases Ves s 1, Sol i 1, *Culex quinquefasciatus* and *Centruroides hentzi*. The remaining proteins were reported on the UniProt database. We considered structures for better visibility of antigenic patches, the parameters for structural quality control for homology models are found in Table 3. To compare the ElliPro results, we chose the main antigen patches with a score higher than 0.7 and more than three residues, taking as reference the epitope of one phospholipase of each group; group A: Ves m 1; group B: Bom p 1; group C: Sol i 1; Group D: Pol d 1 ( Table 2). The constitutional antigenic patches are shown in Figure 2.

**Table 2.** Residues conserved among phospholipases groups with antigenic potential.

Groups of Phospholipases	Residues preserved and antigenic capacity
A (Ves m 1)	T17, N20, N22, D24, L25, Y26, T27, L28, Q29, T30, L31, Q32, N33, H34, P35, E36, F37, K38, K39, K40, T41, I42, T43, R44, P45, D73, N74, Y75, Q118, K119, V121, K122, D123, Y124, K125, I126, S127, M128, A129, N130, R149, Q151, E152, L153, K154, L155, G156, K157
B (Bom p 1)	L98, Y100, P101, I102, V103, K104, C105, K106, V107, K108, S109, T110, I111, L112, C115, K116, E117, Y118, E119, F120, D121, T122, N123, A124, P125, Q126, A:K127
C (Sol i 1)	L52, Y53, N54, S55, F57, Q58, G59, K60, N61, L62, G63, N64, Q65, Q66, S67, C68, Q69, D70, I71, N72, A73, S74, L75, A:P76, F100, V101, Q102, K103, G104, H105, V148, D149, M151, N152, K153, C154, K155, I156, P157, A:N159, L183, I184, N185, K186, T187, P189
D (Pol d 1)	W285, K286, S287, Y288, F289

**Table 3.** Structural quality control parameters for the homology model. The QMEAN4 index has a score is between the range (0–1) that indicates good quality in the model and the GQME index is expressed as a number between 0 and 1. Higher numbers indicate greater reliability.

PARAMETERS							
PROTEINS	Ramachandran favoured	GMQE	QMEAN4	Cβ	All Atom	Solvation	Torsion
Api c 1	97.93%	0.67	-3.02	-2.28	-0.40	0.54	-2.81
Api m 1	96.21%	0.16	-1.58	-0.48	0.53	-1.89	-0.91
Bom p 1	96,27%	0.77	-2.85	-0.97	-1.52	-1.18	-2.40
Bom t 1	97,39%	0.78	-2.85	-0.25	-1.80	-0.74	-2.69
Pol a 1	83,96%	0.33	-6.49	-3.17	-3.20	-2.02	-5.03
Pol d 1	90,41%	0.64	-1.97	0.32	-0.96	0.96	-2.47
Poly p 1	91,19%	0.31	-2.05	0.46	-1.41	0.94	-2.54
Vesp c 1	92,91%	0.96	-1.23	0.18	-1.08	0.53	-1.49
Dol m 1	90,17%	0.81	-1.98	1.16	-0.87	0.86	-2.61
Ves v 1	90,82%	0.78	-1.42	0.98	-0.78	0.82	-1.97
Ves m 1	91,16%	0.88	-1.20	0.87	-0.78	0.95	-1.75
Sol i 1	87,06%	0.56	-4.91	-1.91	-2.20	-0.26	-4.79
Sol i 2	99,12%	0.88	0.23	0.85	1.20	0.80	-0.35
Sol s 2	99,12%	0.76	0.06	0.99	0.85	1.33	-0.76
*Culex quinquefasciatus*	91,07%	0.43	-2.85	-0.53	-1.58	-1.08	-2.36
*Centruroides hentzi*	89.74%	0.64	-4.75	-2.76	-2.82	-0.28	-1.88
*Parasteatoda tepidariorum*	91.60%	0.54	-2.70	-2.24	-1.85	-4.26	-1-69

**Figure 3.**
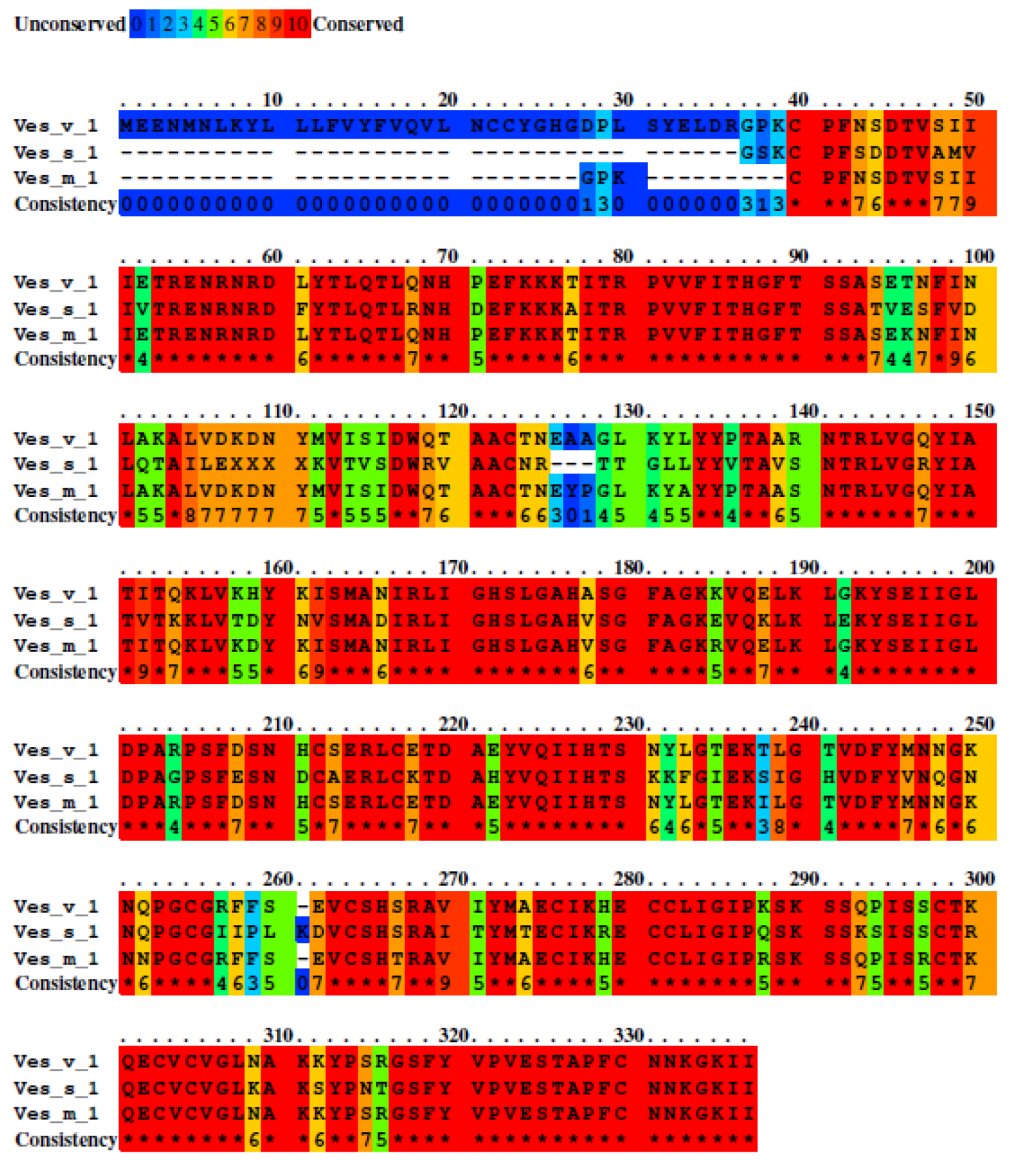
Analysis of group A phospholipases. Unconserved sequence are shown with blue color and high conserved sequence with red color. Moderately conserve sequence are showed with green and orange color. The alignment score was 14,674 with a total of 704 identities residues. The percent sequence identity was 79%.

Phospholipases from group A had a shared identity of 79% between their amino acid sequences ( Figure 3). A total of 704 residues were identified and conserved among the phospholipases analyzed, and for these group, we used Ves m 1 to identify the possible epitopes. We found three common linear antigenic patches and two constitutive antigenic patches with a score greater than 0.7. Also by means of the identity matrix ( Table 4) we can corroborate that their percentages remain high along with other proteins outside of group A.

**Table 4.** Identity matrix of phospholipases.

Allergens	Identity percentages																	
**Api c 1**	100.0	8.86	12.12	10.61	15.84	16.38	15.60	17.14	21.15	15.97	15.38	15.24	17.80	3.45	3.45	7.94	17.29	11.29
**Api m 1**	8.86	100.0	54.48	53.73	15.56	17.01	15.65	17.04	16.18	15.65	14.07	16.30	16.13	12.82	12.82	24.59	14.48	24.07
**Bom p 1**	12.12	54.48	100.0	83.82	18.97	19.83	18.97	19.83	21.37	16.38	16.38	18.97	10.85	10.34	10.34	22.45	16.95	23.26
**Bom t 1**	10.61	53.73	83.82	100.0	18.10	17.24	17.24	16.38	15.38	14.66	14.66	15.52	9.30	6.90	6.90	20.41	15.25	23.26
**Pol a 1**	15.84	15.56	18.97	18.10	100.0	84.05	81.40	52.86	57.05	54.03	53.54	52.72	30.14	14.55	18.18	13.91	30.18	15.38
**Pol d 1**	16.38	17.01	19.83	17.24	84.05	100.0	80.88	52.53	55.52	52.28	52.86	52.04	27.52	19.10	15.73	13.19	27.85	14.39
**Poly p 1**	15.60	15.65	18.97	17.24	81.40	80.88	100.0	57.67	57.95	63.41	64.00	57.58	30.23	18.31	16.90	16.28	28.57	14.06
**Vesp c 1**	17.14	17.04	19.83	16.38	52.86	52.53	57.67	100.0	66.00	71.67	71.00	65.44	33.10	18.52	18.52	9.65	29.12	15.52
**Dol m 1**	21.15	16.18	21.37	15.38	57.05	55.52	57.95	66.00	100.0	59.27	59.33	55.22	29.01	12.28	17.54	13.79	29.97	12.82
**Ves v 1**	15.97	15.65	16.38	14.66	54.03	52.28	63.41	71.67	59.27	100.0	95.67	71.04	31.15	20.24	17.86	11.11	27.33	13.67
**Ves m 1**	15.38	14.07	16.38	14.66	53.54	52.86	64.00	71.00	59.33	95.67	100.0	70.71	33.79	24.07	20.37	10.53	28.17	14.66
**Ves s 1**	15.24	16.30	18.97	15.52	52.72	52.04	57.58	65.44	55.22	71.04	70.71	100.0	32.75	18.52	14.81	12.50	31.23	14.04
**Sol i 1**	17.80	16.13	10.85	9.30	30.14	27.52	30.23	33.10	29.01	31.15	33.79	32.75	100.0	15.56	15.56	11.27	19.62	12.32
**Sol i 2**	3.45	12.82	10.34	6.90	14.55	19.10	18.31	18.52	12.28	20.24	24.07	18.52	15.56	100.0	76.09	9.46	16.92	16.67
**Sol s 2**	3.45	12.82	10.34	6.90	18.18	15.73	16.90	18.52	17.54	17.86	20.37	14.81	15.56	76.09	100.0	10.81	16.92	16.67
**Culex quinquefasciatus**	7.94	24.59	22.45	20.41	13.91	13.19	16.28	9.65	13.79	11.11	10.53	12.50	11.27	9.46	10.81	100.0	12.00	28.39
**Centruroides hentzi**	17.29	14.48	16.95	15.25	30.18	27.85	28.57	29.12	29.97	27.33	28.17	31.23	19.62	16.92	16.92	12.00	100.0	14.38
**Parasteatoda tepidariorum**	11.29	24.07	23.26	23.26	15.38	14.39	14.06	15.52	12.82	13.67	14.66	14.04	12.32	16.67	16.67	28.39	14.38	100.0

**Figure 4.**
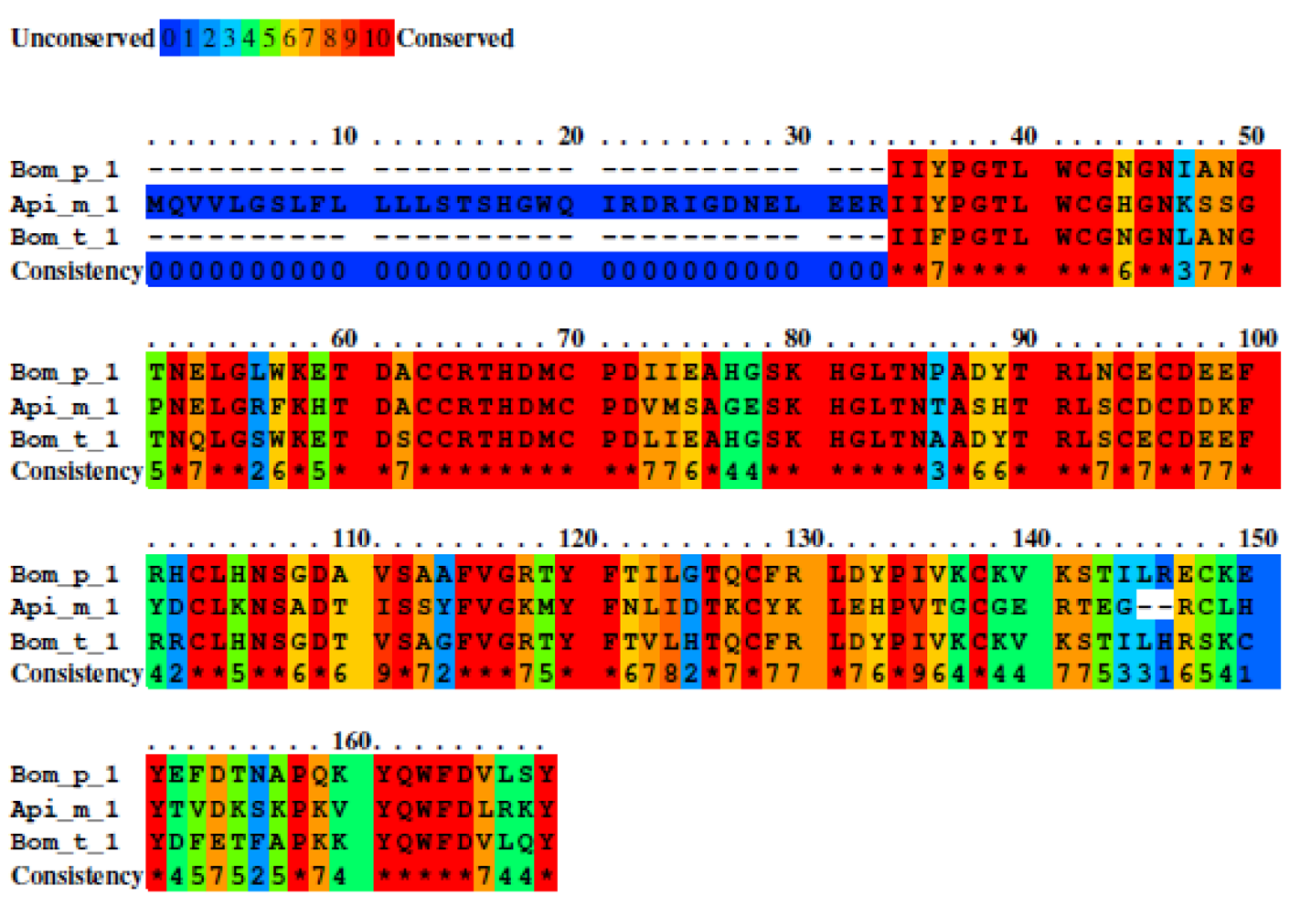
Analysis of group B phospholipases without Api c 1. Three sequences were studied with a total of 439 residues. Unconserved sequence are shown with blue color and high conserved sequence with red color. Middle conserve sequence are showed with green and orange color. A total of 251 residues were identities. The percent sequence identity was 64%.

Group B shares an identity of 35% between their amino acid sequences but when we exclude Api c 1, the identity increases to 64%. In total, 259 identical residues among the sequences were found. We found and included three linear epitopes and two conformational epitopes in Bom p 1 with a score >0.7. ( Figure 4).

Group C, which includes allergens from ants, showed the lowest identity, with only 23% and the highest number of gaps (600 residues missing). Sol i 1 was the protein furthest away from any of the Hymenoptera allergens and appears to be closely related with wasps’ allergens. No common antigenic patches were detected; however, Sol l 1 presents an interesting antigenic patch with 46 residues and a score of 0.711.

**Figure 5.**
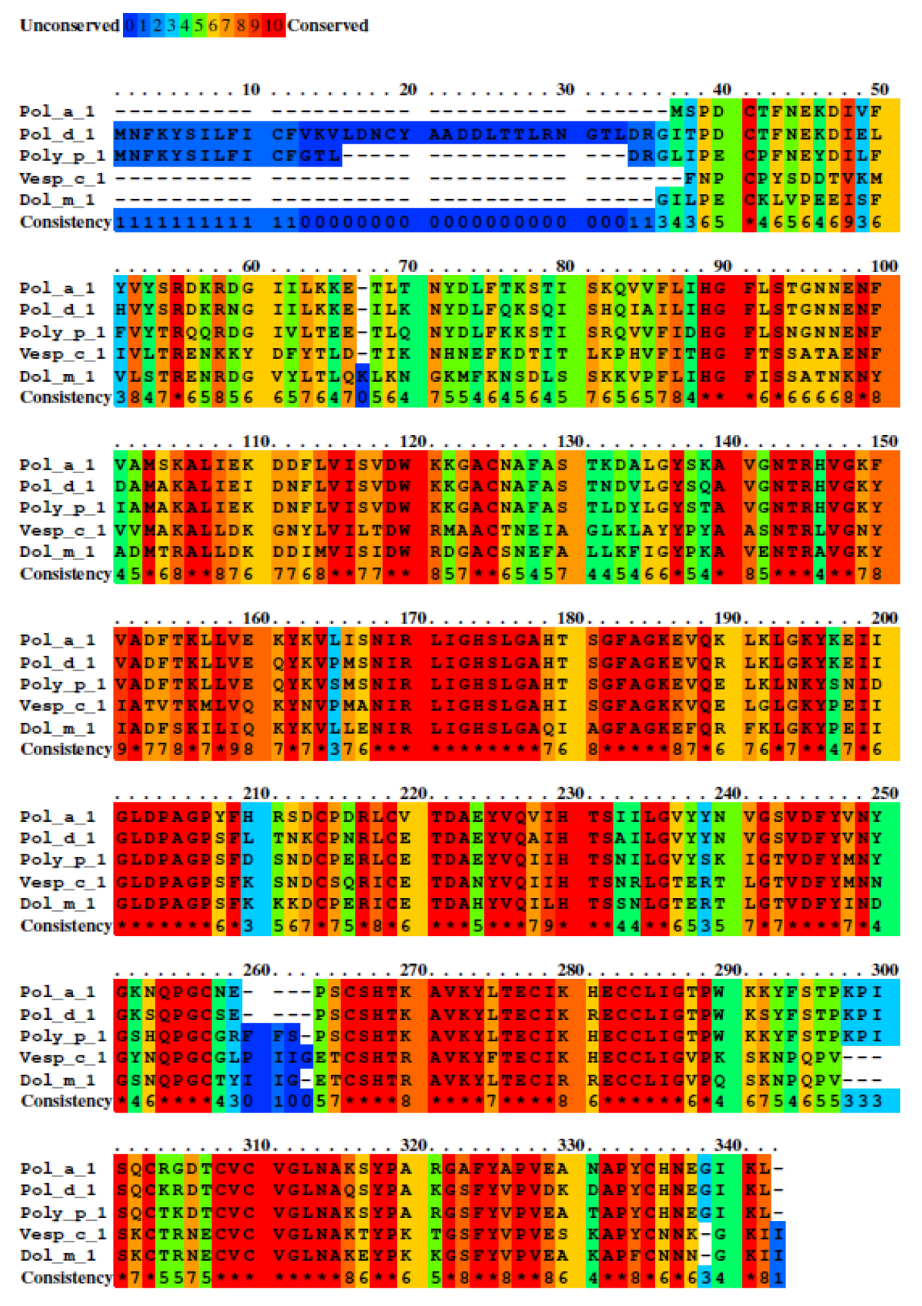
Analysis of group D phospholipases. Five sequences were studied with a total of 1564 residues. Unconserved sequences are shown with blue color and highly conserved sequence with red color. Middle conserve sequence are shown with green and orange colors. The percent sequence identity was 64% with a total of 1916 Aidentities residues.

For group D, 1916 residues exhibit an identity among the five sequences of allergens. This group exhibit a high identity of 64%, the second highest after clade A. For the identification of antigen patches in this group, we used Pol d 1 finding four linear epitopes but only one linear epitope with a valid score ( Figure 5).

## Discussion

Phospholipases A1 and A2 are allergens of insects, which provide a diagnostic benefit for the differentiation of genuine cross-reactivity sensitization. However, the cross-reactivity of this group of allergens has scarcely been holistically explored. In this study, we were able to predict those possible antigenic regions that could explain the cross-reactivity of phospholipases in Hymenoptera through *in silico* analyses.

The 18 amino acid sequences of the allergens were aligned, and a phylogenetic analysis was carried out which yielded four monophylogenetic groups (A, B, C, D). Group A yielded the highest degree of identity among their amino acid sequences 79%. All the allergens of this group belong to the *Vespula* genus, one of the most studied sources of wasp allergens ^7, 19^. In group B (Bom p 1, Bom t 1, Api m 1, Api c 1) two analyses were conducted, the first with the presence of the Api c 1 allergen where a degree of identity of 35% was found and the second without the allergen, where we found a higher degree of identity at 64%. This showed that the alignment of these three species could explain a possible cross-reactivity. Group C (Pol a 1, Pol d 1, Poly p 1, Vesp c 1, Dol m 1.02) showed a level of identity of 64%. However, analysis of conserved and affected residues showed that Group A shares three antigenic regions that could contribute to their cross-reactivity.

IgE against cross-reactive carbohydrate determinants (CCD) is one of the main causes of double positivity and is present in most hymenopteran venom allergens with more frequency in venom from HB and YJ in patients that are allergic to insect bites ^20^. The prevalence of this allergen has been described in more than 20% of patients allergic to honeybee venom; approximately one of four HB poisons and one of 10 YJ venom allergens have been found to be CCDsIgE-positive. The PLA2 structure contains the insect CCDs that are specified by the presence of a 3-core α-1 fucose ^21^. Insect CCD causes 69% at 75% double positive test results for HBV and YJV during allergy diagnosis ^20– 22^. Hemmer *et al*. propose that the Radio Allergo Sorbent Test (RAST) results to OSR pollen appear to be a simple and practicable measure to detect sugar specific IgE in individual sera. This could be useful to discriminate between patients who cross react through CCD and doubly sensitized patients who may require immunotherapy with two poisons.

Currently, CCD-free allergens have been known to allow cross reactivity between proteins to be found without having a double positivity. Ves v 1, Api m 1, Dol m 1, Pol d 1 are allergens that lack cross-reactivity based on CCD and allow diagnoses without interference ^19, 23, 24^. However, it should be clarified that these are mostly of recombinant origin because in its purified natural form possess CCD; for example, Api m 1 of natural origin has CCD and makes diagnosis difficult ^24^. On the other hand, Sol i 1 is the only PLA1 hymenopteran venom known to have CCD, which could make the specific diagnosis of fire ant allergy difficult ^25^.

Research on the allergenic capacity of Hymenoptera allergens has been characterized by individualized studies, with Api m 1, Sol i 1, Pol d 1, Ves m 1 among those most studied so far, but the possible cross-reactivity between phospholipase allergens A1 and A2 has not been holistically evaluated ^2, 24, 26^. No cross-reactivity between *A. mellifera, S. invicta* and *V. vulgaris* was detected, which supports our results, since there was no relationship between these allergens. However, when analyzed along with other allergens, it was observed that a certain degree of identity is maintained between these two proteins, suggesting a possible cross reactivity without CCD. In the Table 5 we can see the presence or absence of CCD and comparison between reported clinical cross-reactivities and obtained cross-reactivities.

**Table 5.** Presence or absence of CCD and comparison between reported clinical cross-reactivities and obtained cross-reactivities.

Allergens	Absence of CCD	Clinical cross- reactivity Demonstrated	cross reactivities obtained	References
Group A				
Ves m 1	No report	With Dol m 1, Ves v 1	This Group has to identity of 79%	King, Te Piao, *et al.* “Yellow Jacket Venom Allergens, Hyaluronidase and Phospholipase: Sequence Similarity and Antigenic Cross-Reactivity with Their Hornet and Wasp Homologs and Possible Implications for Clinical Allergy.” *Journal of Allergy and Clinical Immunology*, vol. 98, no. 3, 1996, pp. 588–600, doi: 10.1016/S0091-6749(96)70093-3.
Ves s 1	No report	_____		
Ves v 1	Absence	With Dol m 1, Pol d 1		
Group B				
Api c 1	No report	_____	This Group has to identity of 64% without api c 1	- Jakob, Thilo, Julian Köhler, *et al.* “Comparable IgE Reactivity to Natural and Recombinant Api m 1 in Cross-Reactive Carbohydrate Determinant-Negative Patients with Bee Venom Allergy.” *Journal of Allergy and Clinical Immunology*, vol. 130, no. 1, 2012, pp. 276–78, doi:: 10.1016/j.jaci.2012.03.048. - Müller, U, *et al.* *IgE to Recombinant Allergens Api m 1 , Ves* *v 1 , and Ves v 5 Distinguish Double Sensitization from* *Crossreaction in Venom Allergy*. 2012, doi: 10.1111/j.1398-9995.2012.02847.x.
Api m 1	Present	With Ves v 1, Ves s 1		
Bom p 1	No report	_____		
Bom t 1	No report	_____
Group C				
Sol i 1	Present	Poly p 1, Ves m 1, Ves v 1, Dol m 1	This Group has to identity of 23%	- Perez-Riverol, Amilcar, *et al.* “Venoms of Neotropical Wasps Lack Cross-Reactive Carbohydrate Determinants Enabling Reliable Protein-Based Specific IgE Determination.” *Journal* *of Allergy and Clinical Immunology*, vol. 141, no. 5, 2018, pp. 1917–20, doi:10.1016/j.jaci.2017.12.990. - Hoffman, Donald R., *et al.* “Sol i 1, the Phospholipase Allergen of Imported Fire Ant Venom.” *Journal of Allergy* *and Clinical Immunology*, vol. 115, no. 3, 2005, pp. 611–16, doi: 10.1016/j.jaci.2004.11.020.
Sol i 2	No report	_____		
Sol s 2	No report	_____		
Group D				
Pol d 1	Absence	Poly p 1, Ves s 1	This Group has to identity of 64%	- Perez-Riverol, Amilcar, Luís Gustavo Romani Fernandes, *et al.* “Phospholipase A1-Based Cross-Reactivity among Venoms of Clinically Relevant Hymenoptera from Neotropical and Temperate Regions.” *Molecular* *Immunology*, vol. 93, Elsevier, 2018, pp. 87–93. - Monsalve, R. I., *et al.* “Component-Resolved Diagnosis of Vespid Venom-Allergic Individuals: Phospholipases and Antigen 5s Are Necessary to Identify Vespula or Polistes Sensitization.” *Allergy: European Journal of Allergy and Clinical* *Immunology*, vol. 67, no. 4, 2012, pp. 528–36, doi:, doi: 10.1111/j.1398-9995.2011.02781.x
Pol a 1	No report	Poly p 1		
Poly p 1	No report	Sol I 1, Pol d 1, Pol a 1, Ves v 1		
Dol m 1	Absence	Ves v 1, Ves m 1		
Vesp c 1	No report	_____		

**Figure 6.**
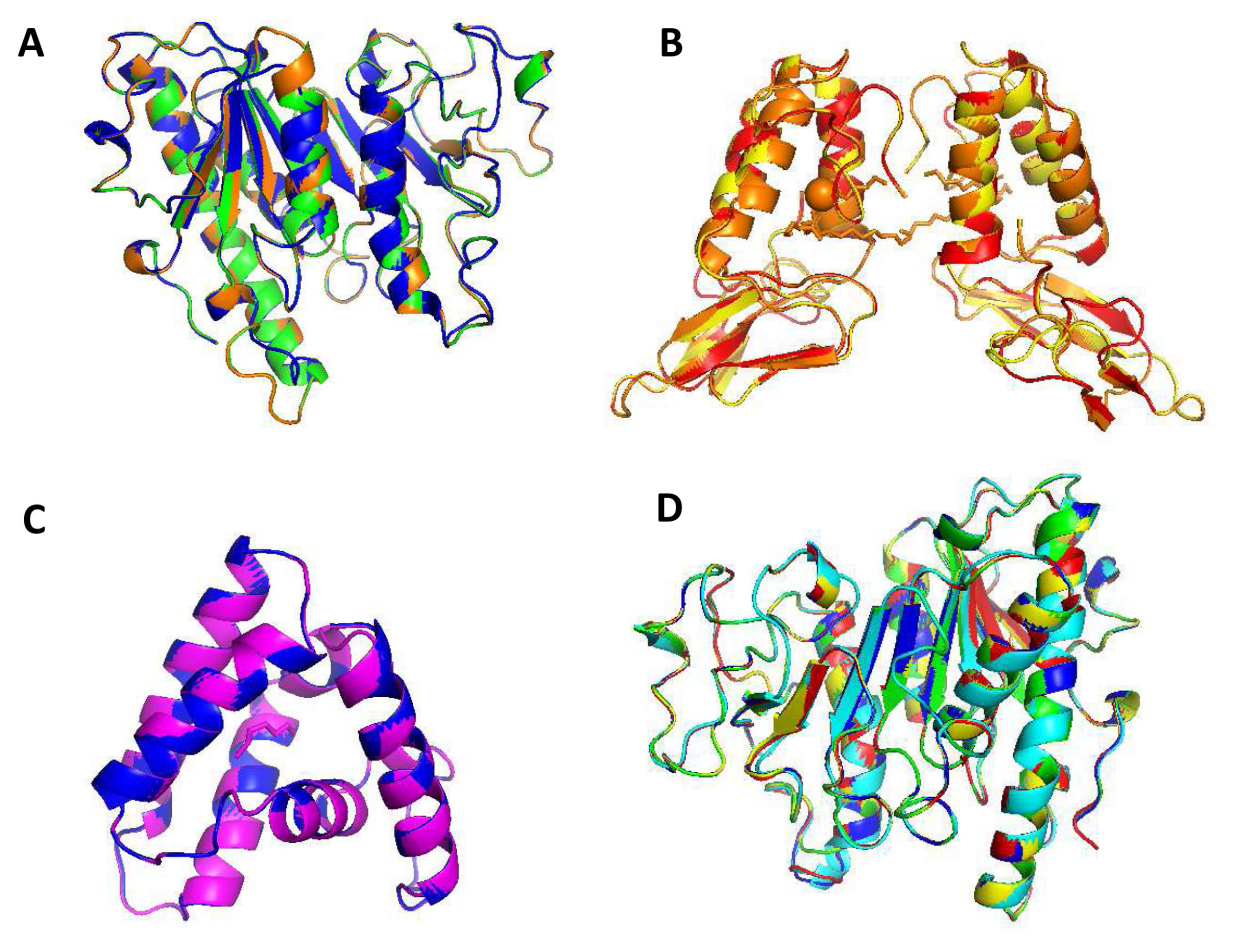
Overlapping of phospholipases. ( **A**) Ves v 1 in green, Ves m 1 in orange and Ves s 1 in blue. ( **B**) Api c 1, Api m 1, Bom p 1, Bom t 1, *Centruroides hentzi* and *Parasteatoda tepidariorum*, but we only found structural homology in Api m 1 of orange color, Bom p 1 of yellow color and Bom t 1 of red color. ( **C**) Sol i 1, Sol i 2, Sol s 1, and *Culex quinquefasciatus* but we only found structural homology in Sol i 2 of a magenta color, Sol s 1 of blue color. ( **D**) Pol a 1 of green color, Pol d 1 of yellow color, Poly p 1 of blue color, Dol m 1 of cyan color and Vesp c 1 of red color.

Group A (Ves m 1, Ves s 1 and Ves v 1) being the most representative, the cross reactivity between *Vespula* spp. is strong due to the similarities in the composition of the poison and the structure of the individual allergens ^27^. Different studies evaluate the identity of the yellow jackets; for example, a 1996 study reported that Ves v 1 had 95% identity with Ves m 1 and both yellow jacket phospholipases have about 67% sequence identity with the hornet protein Dol m 1 ^7^. Other authors demonstrated that Ves v 1 also shows an identity of 54% with Poly p 1, it being the lowest among the allergens studied and a study carried out in Spain with 59 previously diagnosed allergic patients with an allergy to vespid found that there could be a double sensitization between Ves v 1 and Pol d 1 because in 31% of patients they could not be clearly defined as sensitized only to Vespula or Polistes ^28, 29^. Consequently, the different *Vespula* poisons react strongly in a crossed manner, which would explain the high degree of identity found in the study (Group A (79%)). Of the three proteins, only Ves v 1 has been described as a CCD allergen, showing that this interaction between the *Vespula* phospholipases could be CCD-independent and related only by protein structure ^19^. The quaternary structure of the three *Vespula* phospholipases is also very similar, suggesting the possibility of present both linear and conformational epitopes ( Figure 6A). Therefore, we suggested that fragment inhibition studies be carried out to identify the possible antigenic peptide described in this study.

Group B showed a degree of identity of 35%, however, in the analysis, we found that if we performed the alignment without the Api c 1 allergen, the degree of conservation between Api m 1, Bom p 1 and Bom t 1 increased to 64%. So far, we have found no more information about the possible cross reactivity in these allergens. In this group, Api m 1 is the most characterized allergen; it contains the cross-reactive carbohydrate (CCD) determinants of insects that are defined by the presence of a 3-core α-1 fucose ^30^.

For years, the detection of Api m 1 CCD challenges the differentiation of HB and YJ allergy. However, *in vitro* detection of immunoreactive sIgE from these insects showed double positivity in up to 59% of the patients ^24^. PLA2s possess important venom allergens in other members of the genus *Apis* and *Bombus* that have been shown to have homology. *A. cerena* (Api c 1) have been little explored but have been described as having high identity levels with other phospholipases, like *A. mellifera* (95%) ^26^. In our study, we observed that when comparing the sequences of these phospholipases with those of the genus *Bombus*, that identity was not preserved since the identity we found was very low and when excluding it from the alignment, the sequences were more conserved ^31^. Studies conducted on the genus *Bombus* found that the primary sequences of Bom t 1 and Api m 1 have an identity of 53% and their three-dimensional structures show conserved low protein surfaces ^32^. However, the allergens selected from group B in our study showed a high conservation and structural homology leading to possible cross-reactivity ( Figure 6B).

As for Group C, we highlight that it was the only group that included phospholipases A1 and A2 in the clade, so a low identity was expected. We found that the ant phospholipases Sol i 1, Sol i 2 and Sol s 2 showed a degree of alignment identity with the other phospholipases in the primary sequences of 23%. This low identity is not enough to explain cross-reactivity *in silico*, even though allergen Sol i 1 has been extensively analyzed and other studies suggest that it may have a possible reactivity with the *Centruroides* species ^33, 34^.

The phylogenetic analyzes reported in this study revealed that Sol i 1 is the most divergent member among the currently identified hymenopteran venom group PLA1. As noted, Sol i 1 is in a group (group C) completely isolated from the clade consisting of wasp allergenic PLA1 (group A) and showed no structural homology ( Figure 6C). Furthermore, in multiple alignments, the fire ant exhibits the lowest level of sequence identity. However, studies have shown cross-reactivity between Sol i 1 and its wasp counterparts with amino acid sequence identity levels of 38% with Ves m 1, 36% with Ves v 1, 40% with Dol m, 1.35% with Pol d. 1.36% with Poly p 1 ^35^. However, a recent study suggests that peptide-based cross-reactivity between Sol i 1 and PLA1 of Polistinae wasps does not occur because the alignments and the phylogenetic and structural analyzes showed that it is an allergen further from its counterparts, in addition to possessing the lowest level of identity among the sequences studied, with 36%, and the highest RMSD value with 0.172 ^29^.

Several works have attempted to demonstrate cross-reactivity between A1 phospholipases ^29, 36^. The cross-reactivity based on PLA1 of the venoms of eight hymenoptera was analyzed and it was described that the identity of the primary sequence of Poly p 1 was conserved in 36% with Sol i 1, 74% with Pol d 1 and 71 % with Pol a 1. In our study no relationship was found between Poly p 1 with Sol i 1. However, group D, where we found the different species of Polistes (Pol a 1 and Pol s 1), Poly p 1, Dol m 1 and Vesp c 1, showed a high degree of identity of 64% and structure homology ( Figure 6D), enough to explain cross-reactivity ^29^. An attempt was made to look for cross reactivity between Dol m 1, Ves v 1 and pol a 1 with mice; partial cross-reactivities in the T-cell epitopes of homologous vespid allergens was found, which supports our findings ^7, 29, 36^.

Of the species chosen, three non-allergenic phospholipases (Centruroides *hentzi, Parasteatoda tepidariorum, and Culex quinquefasciatus*) were taken to adjust the phylogenetic analysis, so as the results were produced, we observed that these phospholipases separated into two clades showing some affinity for some phospholipases allergens.

A study identified allergens in the venom of common striped scorpions. Eleven patients with scorpion venom allergy were assessed, where four patients had a history of anaphylaxis (with positive skin test responses) to imported fire ant venom (IFA) and at least two other had a history of large local reactions, suggesting that there could be a cross reactivity between proteins of these insects; this association would be clinically relevant ^29^. This shows that despite not being described as allergens, it is necessary to carry out studies to verify their capacity to trigger sensitization.

Bioinformatic studies are high impact tools of great importance. Currently they are recognized as the first step to conducting an investigation, since they are *in silico* analyzes that facilitate a possible approximation to expected results, allow predictions or models, and serve as the basis for the emergence of large projects. In our study, we show possible antigenic regions involved in cross-reactivity between phospholipases A1 and A2, based on what was found with the use of *in silico* analysis we can say that they are proteins with a high degree of identity and that three antigenic regions were found, which would explain possible co-sensitization.

It is also necessary to note that our study has some weaknesses that could explain the lack of cross-reactivity between the allergens evaluated; In the case of phospholipases, we model its tertiary structure based on other homologous proteins since its tertiary structure is unknown, however in-silico constructions are not exact and it is possible that its natural form is different from what we propose. In the same direction, the epitopes require further confirmation through studies in biological models, in vivo, in vitro and experimental.

## Conclusion

Potential antigenic sites were identified for the generation of cross-reactivity between the phospholipases analyzed in this study. The identity between these proteins of different species is relatively high, which shows that cross-reactivity between them is possible and their frequency in most cases can be high. These studies support diagnostic testing by component studies for venom allergy and the need to carry targeted mutagenesis tests is important to confirm their relevance in the allergenic capacity of phospholipases.

## Data availability

All data underlying the results are available as part of the article and no additional source data are required.
