# Supplementary material for: In silico analysis of cross reactivity among phospholipases from Hymenoptera species
Source: F1000Res. 2021 Mar 29;10:2. Originally published 2021 Jan 5. [Version 2] doi: 10.12688/f1000research.27089.2 (PMC8129813; doi:10.12688/f1000research.27089.2)
Supplement: Peer review history

# Peer review: Reviewer response for version 2

**DOI:** 10.5256/f1000research.55581.r82410

**Published:** 2021-05-17

**Authors:** Jose F. Cantillo

**Affiliations:** 1 Department of Microbiology and Immunology, University of Illinois at Chicago, Chicago, IL, USA

In the second version of the manuscript: "In silico analysis of cross reactivity among phospholipases from Hymenoptera species" the authors have made changes as suggested and included clarifications. However, some of the questions were addressed in the responses but I do not find the changes in the manuscript. Overall, the explanations and clarifications they provided are ok. For some of the questions, I think the responses were not adequately resolved. However, once all of the changes that were cited in the responses are included in the final version, I consider that the paper would be adequate for indexing.

Is the work clearly and accurately presented and does it cite the current literature?

Partly

If applicable, is the statistical analysis and its interpretation appropriate?

Not applicable

Are all the source data underlying the results available to ensure full reproducibility?

Partly

Is the study design appropriate and is the work technically sound?

Partly

Are the conclusions drawn adequately supported by the results?

Partly

Are sufficient details of methods and analysis provided to allow replication by others?

Partly

Reviewer Expertise:

Immunology, allergy, recombinant allergens, mosquito and house dust mite allergy, somatic hypermutation and class switch recombination.

I confirm that I have read this submission and believe that I have an appropriate level of expertise to confirm that it is of an acceptable scientific standard.

# Peer review: Reviewer response for version 2

**DOI:** 10.5256/f1000research.55581.r82409

**Published:** 2021-04-19

**Authors:** Anna Pomés

**Affiliations:** 1 Basic Research, Indoor Biotechnologies, Inc., Charlottesville, VA, USA

The authors have improved the manuscript, including the addition of 2 new Tables. However, some answers to the reviewer’s comments were not implemented in the text and they should be.

 Regarding the comment: “Answer: we accept the observation and proceed to change constitutive antigenic parch from conformational epitopes in the second version”, **conformational antigenic patches** (instead of constitutional or constitutive epitopes) should be used all over the manuscript.

 Legend to figure 2:  Sol i 1 (not Sol I 1). “the Vespid basal is sequence” needs to be corrected.

 Regarding the answer to this point:

 “9. Discussion, third paragraph: “double positivity” needs to be explained (69% at 75% is not clear). What is OSR? (spell out).”

 The authors responded but did not apply changes to the discussion. For example:  double positivity of IgE results to allergen proteins and to the CCDs. Also, the OSR needs to be spelled out in the text, unless the abbreviation was explained somewhere else.

 The authors improved the manuscript adding Tables 4 and 5.  Table 5 needs some correction of the expression (used in several cells): “This Group has to identity of 64% without api c 1” to say: “This Group shares 64% amino acid identity, except Api c 1” (Api in capital).

 Regarding their answer to Minor comments, the terms were explained in the answer to the reviewer but should be explained in the manuscript as well.

Is the work clearly and accurately presented and does it cite the current literature?

Partly

If applicable, is the statistical analysis and its interpretation appropriate?

Not applicable

Are all the source data underlying the results available to ensure full reproducibility?

Yes

Is the study design appropriate and is the work technically sound?

Partly

Are the conclusions drawn adequately supported by the results?

Partly

Are sufficient details of methods and analysis provided to allow replication by others?

Partly

Reviewer Expertise:

Allergen structure/function and antigenic determinants

I confirm that I have read this submission and believe that I have an appropriate level of expertise to confirm that it is of an acceptable scientific standard.

# Peer review: Reviewer response for version 1

**DOI:** 10.5256/f1000research.29923.r76667

**Published:** 2021-02-03

**Authors:** Jose F. Cantillo

**Affiliations:** 1 Department of Microbiology and Immunology, University of Illinois at Chicago, Chicago, IL, USA

**Major comments:**

 The manuscript presented by Emiliani *et al.* presents a theoretical analysis of cross-reactivity among Hymenoptera-derived phospholipases. 18 sequences were retrieved and splitted in 4 clades after phylogenetic analysis. Prediction of IgE-cross reactive epitopes was performed using a bioinformatic tool and multiple alignment. There are some considerations that the authors must address to provide a more solid argument, and further analysis must be performed.

- Why did the authors used ElliPro server and not another one? Is there any advantage of this tool compared to others?
- Prediction of antigenic regions should be performed using different tools and individual analysis of accessibility, hydrophobicity, etc. And then compared the results to provide the “most probable” antigenic region.
- The authors use the term “constitutional antigenic patches”. What does this mean? Is it a concept used by the bioinformatic tool? If so, they must explain what this is about.
- Comparisons between aa sequences result in percentages of identity as a way to express the level of conservation or homology. However, proteins like phospholipases have variable molecular weights, which means that they aren’t similar in the whole length of their sequences. The authors must make clear that a portion of each protein is used to make the statements related to the identities and that the analysis is restricted to these areas.
- Consequently, was the homology model made for the whole Ves s 1, Sol I 1, *C. quinquefasciatus* and *C. hentzi* phospholipases as whole proteins or just a portion of them?
- In the methods section, which templates were used by SWISS MODEL to predict the 3D model?
- How was the superimposition of 3D structures presented in figure 5 performed?. The approach should be indicated in the methods.
- The discussion is too long given the presented results. Sometimes the information provided is repetitive. For example, the second paragraph of the discussion is essentially previously mentioned in the results section and can be eliminated.
- The third paragraph in the discussion section refers to carbohydrate determinants, which are not even superficially explored in this study. Although the observation referred to CCD can be mentioned in this manuscript, is not necessary to discuss this issue too much since there are no analysis for this matter in this paper.
- A similar evaluation of all the information provided in the discussion should be done. It is difficult to understand what the authors are trying to argue and how this is closely related to the actual results. A shortened and more precise discussion could greatly improve the manuscript.

**Minor comments:**

- English and grammar needs to be revised.  Many sentences need to be re-written to better disclose the message that the authors are trying to say, for example:
  - Allergic immune response from hymenopteran allergens has been studied in detail due to a high incidence of sting reactions to these insects…
  - A closed relationship among phospholipase allergens as shown, formed four nodes with a high phylogenetic relationship among them…
- A better use of “comas” and “periods” must be done in order to improve the writing as well. For example:
  Molecular, structural, and immunological characterization of hymenopteran venom allergens is advanced, in total 75 allergens from 31 different species have been explored, and since phospholipases are a family of allergens with clinical and biological relevance, some proteins belonging to this order such as hyaluronidase and antigen V are also considered relevant to sensitization to this allergenic
- In figure 3, what does “identities residues” mean?.
- If a total of 704 residues were identified and conserved among the phospholipases, why is figure 3 showing around 330 only?.
- We built four 3D models of the 18 phospholipases Ves s 1, Sol i 1, Culex quinquefasciatus and Centruroides hentzi… are you referring to “18” or “4”proteins?.
- Figures 3, 4 and 5 only show the multiple sequence alignments, but not the identified epitopes as the authors are pointing out. Please correct.
- In group B (Bom p 1, Bom t 1, Api m 1, Api c 1) two analyses were conducted, the first with the presence of the Api c 1 allergen where a degree of identity of (35%) was found and the second without the allergen… why are you using parenthesis?

Is the work clearly and accurately presented and does it cite the current literature?

Partly

If applicable, is the statistical analysis and its interpretation appropriate?

Not applicable

Are all the source data underlying the results available to ensure full reproducibility?

Partly

Is the study design appropriate and is the work technically sound?

Partly

Are the conclusions drawn adequately supported by the results?

Partly

Are sufficient details of methods and analysis provided to allow replication by others?

Partly

Reviewer Expertise:

Immunology, allergy, recombinant allergens, mosquito and house dust mite allergy, somatic hypermutation and class switch recombination.

I confirm that I have read this submission and believe that I have an appropriate level of expertise to confirm that it is of an acceptable scientific standard, however I have significant reservations, as outlined above.

# Response

**Published:** 2021-03-10

**Authors:** Andrés Sánchez

**Affiliations:** Corporation Universitary Rafael Nuñez, Colombia

Jose F. Cantillo

 Department of Microbiology and Immunology, University of Illinois at Chicago, Chicago, IL, USA 

 Dear reviewer.

 We have addressed all the suggestions, below are the comments.

**Answer at the comments:**

 1. Why did the authors used ElliPro server and not another one? Is there any advantage of this tool compared to others?

 Answer: it is a tool that provides greater robustness to predictive models and has been shown to be a better server for conformational epitopes.

 - J Ponomarenko, H H Bui, W Li, N Fusseder, P E Bourne, A Sette, B Peters, ElliPro: a new structure-based tool for the prediction of antibody epitopes, BMC Bioinf. 9 (2008) 514.

 2. Prediction of antigenic regions should be performed using different tools and individual analysis of accessibility, hydrophobicity, etc. And then compared the results to provide the “most probable” antigenic region.

 Answer: Given the robustness of the program, we do not consider the use of other tools.

 3. The authors use the term “constitutional antigenic patches”. What does this mean? Is it a concept used by the bioinformatic tool? If so, they must explain what this is about.

 Answer: in the second version we will change the term to conformational epitopes for clarity.

 4. Comparisons between aa sequences result in percentages of identity as a way to express the level of conservation or homology. However, proteins like phospholipases have variable molecular weights, which means that they aren’t similar in the whole length of their sequences. The authors must make clear that a portion of each protein is used to make the statements related to the identities and that the analysis is restricted to these areas.

 Answer: the PRALINE server makes an adjustment according to the length, based on the coverage between the proteins. The areas that are being conserved will be shown in the images.

 5. Consequently, was the homology model made for the whole Ves s 1, Sol I 1, C. quinquefasciatus and C. hentzi phospholipases as whole proteins or just a portion of them?

 Answer: Yes, the whole protein model was made.

 6. In the methods section, which templates were used by SWISS MODEL to predict the 3D model?

 Answer: The template that SWISS MODEL uses to predict tertiary structures is based on other homologous proteins with known tertiary structure. The server shows which is the base protein that is being taken for the creation of the new protein. This proteins will be show in the second version.

 7.  How was the superimposition of 3D structures presented in figure 5 performed?. The approach should be indicated in the methods.

 Answer: it was done using the matchmaker tool of the Pymol program.

 8. The discussion is too long given the presented results. Sometimes the information provided is repetitive. For example, the second paragraph of the discussion is essentially previously mentioned in the results section and can be eliminated.

 Answer: we consider it important to make a short summary of the results for a better understanding of the discussion.

 9. The third paragraph in the discussion section refers to carbohydrate determinants, which are not even superficially explored in this study. Although the observation referred to CCD can be mentioned in this manuscript, is not necessary to discuss this issue too much since there are no analysis for this matter in this paper.

 Answer: we preserve the written information from CCD because the information was suggested by the editor and another reviewer.

 9. A similar evaluation of all the information provided in the discussion should be done. It is difficult to understand what the authors are trying to argue and how this is closely related to the actual results. A shortened and more precise discussion could greatly improve the manuscript.

 Answer: thanks we will take it into account for the second version

**Minor comments:**

 1. English and grammar needs to be revised.  Many sentences need to be re-written to better disclose the message that the authors are trying to say, for example:

 - Allergic immune response from hymenopteran allergens has been studied in detail due to a high incidence of sting reactions to these insects…

  - A closed relationship among phospholipase allergens as shown, formed four nodes with a high phylogenetic relationship among them…

 - A better use of “comas” and “periods” must be done in order to improve the writing as well. For example:

 Molecular, structural, and immunological characterization of hymenopteran venom allergens is advanced, in total 75 allergens from 31 different species have been explored, and since phospholipases are a family of allergens with clinical and biological relevance, some proteins belonging to this order such as hyaluronidase and antigen V are also considered relevant to sensitization to this allergenic.

 Answer: English grammar is reviewed, as well as the use of points and commas.

 2.  In figure 3, what does “identities residues” mean?

 Answer: They could also be called conserved residues, these correspond to the amino acids that are being recognized as causing cross-reactivity, showing high identities between the different sequences analyzed.

 3. If a total of 704 residues were identified and conserved among the phospholipases, why is figure 3 showing around 330 only?

 Answer: the PRALINE tool used for the alignment shows the number of residues taken into account for the analysis, what was done was to calculate how many residues of that total were conserved in those analyzed sequences. Thus, in figure 3, 934 of which 704 were conserved were taken into account in the analysis, this figure corresponds to 79% of the total residues.

 4. We built four 3D models of the 18 phospholipases Ves s 1, Sol i 1, Culex quinquefasciatus and Centruroides hentzi… are you referring to “18” or “4”proteins?

 Answer: we will refer only to the 4 proteins that are named. They are those that failed to overlap due to the absence of structural homology.

 5. Figures 3, 4 and 5 only show the multiple sequence alignments, but not the identified epitopes as the authors are pointing out. Please correct.

 Answer: we will select the linear epitope within the figure.

 6. In group B (Bom p 1, Bom t 1, Api m 1, Api c 1) two analyses were conducted, the first with the presence of the Api c 1 allergen where a degree of identity of (35%) was found and the second without the allergen… why are you using parenthesis?

 Answer: It is fixed for the second version.

# Peer review: Reviewer response for version 1

**DOI:** 10.5256/f1000research.29923.r76669

**Published:** 2021-01-18

**Authors:** Anna Pomés

**Affiliations:** 1 Basic Research, Indoor Biotechnologies, Inc., Charlottesville, VA, USA

**General comments:**

 The manuscript by Emiliani *et al.* reports an *in silico* analysis of cross-reactivity among phospholipases from Hymenoptera species. Four groups or clades are identified and further analyzed by sequence alignments and epitope prediction.  However, the potential IgE cross-reactivity between pairs of proteins within and between different clades are not clear from the analysis. The discussion needs to be revised to explain better their results compared to clinical observations reported in the literature.

 First paragraph from results:  From Table 1 it seems that 15 allergenic and 3 non allergenic sequences were selected. The way it is phrased it seems as if 18 allergenic sequences were selected. The numbers of species do not add up to 18 (5 bees, 6 wasps, 3 ants, 3 other = 17). Are yellow jackets and hornets considered wasps? If so there should be 8 wasps, and 4 bees.

 Table 3: The range 0-1 indicated for QMEAN4 index in the Legend to table 3 does not correspond to the range of values -6.49 to 0.23 in the Table. “has a score is” needs correction.

 The authors should explain what do they mean by “constitutional” or “constitutive” antigenic patches (page 5). Do the authors mean “conformational or discontinuous epitopes”? (not “discontinues” as in page 6).

 Legend to figure 2:  Sol i 1 (not Sol I 1). “the Vespid basal is sequence” does not seem correct.

 Page 5, 3 ^rd^ paragraph: how can 704 residues be conserved among 337 residues per sequence (in the alignment of Figure 3).  The authors should explain where the number 704 comes from. Similarly, the same applies to 259 and 1916 residues from sequences that are shorter in Figures 4 and 5, respectively. Usually, percent of identity between two sequences is a better way to express homology than saying the total residues that were conserved for all the sequences (this is what the authors seem to be presenting).  A better way that the authors could use to show homologies among several sequences, is an Percent Identity Matrix, which shows all the percentages of identity between pairs of proteins.

 Sol i 1 in page 6, second paragraph.

 Linear and continuous epitopes are the same. In page 8, first paragraph:  do the authors mean to use linear and continuous in the same line?

 Discussion, end of paragraph 2:  Do groups A and D share antigenic areas? (what are “affected residues”?).  If so, the explanation is not clear, and not shown in the results.  Maybe an overlay of a representative molecule from each of the two groups showing the areas that are shared in green could be helpful in the results section to illustrate this (and blue and yellow areas in the respective molecules).

 Discussion, third paragraph:  “double positivity” needs to be explained (69% at 75% is not clear). What is OSR? (spell out).

 The discussion is hard to follow, maybe in part because it is not clear from the results if cross-reactivity is expected or not among different species. It might be interesting to have a table in the results section showing the 4 groups with their proteins (in first row and first column) and indicating if clinical cross-reactivity has been observed (also CCD presence or absence if it applies), next to expected cross-reactivities from the results.

**Minor comments:**

- English grammar needs revision.  For example, in Abstract: “phospholipase allergens”, “18 amino acid sequences”, “shared an amino acid sequence identity”.
- Some terms need explanation for the readers to understand (for example: “empty spaces”, “length of the branches”, “nodes” in first paragraph, page 4).
- Page 3, 1st column, paragraph 2, line 14: “sensitization to this allergenic.” Needs to be completed (allergenic source?).
- Page 3, second column, 3rd paragraph, line 10:  sentence with “areas” is vague. Does “area” mean epitopes or something else? Line 11: “Various studies have been carried out…”
- Page 5, first paragraph: *P. paulista*. Other species names should be in italics as well all over the manuscript (i.e. Legend to Figure 6).
- Page 5, 1 ^st^ paragraph, last line: analyses.
- Legends to Figures 3, 4 and 5 need corrections (highly conserved, middle conserved sequences, were identified).
- Verb “Consisting” can be removed from Legend to figure 6.

Is the work clearly and accurately presented and does it cite the current literature?

Partly

If applicable, is the statistical analysis and its interpretation appropriate?

Not applicable

Are all the source data underlying the results available to ensure full reproducibility?

Yes

Is the study design appropriate and is the work technically sound?

Partly

Are the conclusions drawn adequately supported by the results?

Partly

Are sufficient details of methods and analysis provided to allow replication by others?

Partly

Reviewer Expertise:

Allergen structure/function and antigenic determinants

I confirm that I have read this submission and believe that I have an appropriate level of expertise to confirm that it is of an acceptable scientific standard, however I have significant reservations, as outlined above.

# Response

**Published:** 2021-03-10

**Authors:** Andrés Sánchez

**Affiliations:** Corporation Universitary Rafael Nuñez, Colombia

Anna Pomés.

 Indoor Biotechnologies, Inc., Charlottesville, United States.

 Dear reviewer.

 We have addressed all the suggestions, below are the comments.

**Answer at the comments:**

 1. First paragraph from results:  From Table 1 it seems that 15 allergenic and 3 no allergenic sequences were selected. The way it is phrased it seems as if 18 allergenic sequences were selected. The numbers of species do not add up to 18 (5 bees, 6 wasps, 3 ants, 3 other = 17). Are yellow jackets and hornets considered wasps? If so there should be 8 wasps, and 4 bees.

 Answer: First paragraph of the results an error is evidenced, because the correct phrase is how you put it. We selection 15 sequences allergenic and 3 nor allergenic. The sum of the species was organized bad, the really are 2 bees, 2 bumblebee, 6 wasp, 2 hornets, 3 ant and 3 no allergenic, respectively.

 2. Table 3: The range 0-1 indicated for QMEAN4 index in the Legend to table 3 does not correspond to the range of values -6.49 to 0.23 in the Table. “has a score is” needs correction.  

 Answer: the global score is originally in a rank [0, 1] being one a good. Default, the transformed in Z score from relate then to what we would expect from X ray structure of high resolution.

 3.  The authors should explain what do they mean by “constitutional” or “constitutive” antigenic patches (page 5). Do the authors mean “conformational or discontinuous epitopes”? (not “discontinues” as in page 6).

 Answer: we accept the observation and proceed to change constitutive antigenic parch from conformational epitopes in the second version.

 4. Legend to figure 2:  Sol i 1 (not Sol I 1). “the Vespid basal is sequence” does not seem correct.

 Answer: it was correct the name of the antigen: Sol I 1, both in the legend as in the paragraph 6.

 5. Page 5, 3rd paragraph: how can 704 residues be conserved among 337 residues per sequence (in the alignment of Figure 3).  The authors should explain where the number 704 comes from. Similarly, the same applies to 259 and 1916 residues from sequences that are shorter in Figures 4 and 5, respectively. Usually, percent of identity between two sequences is a better way to express homology than saying the total residues that were conserved for all the sequences (this is what the authors seem to be presenting).  A better way that the authors could use to show homologies among several sequences, is an Percent Identity Matrix, which shows all the percentages of identity between pairs of proteins.

 Answer: the PRALINE tool used for the alignment shows the number of residues taken into account for the analysis, what was done was to calculate how many residues of that total were conserved in those analyzed sequences. Thus, in figure 3, 934 of which 704 were conserved were taken into account in the analysis, this figure corresponds to 79% of the total residues and similarly applies to residues 259 and 1916 of figures 4 and 5. The matrix Percentage identity is included in the second version.

 6. Sol i 1 in page 6, second paragraph.

 Answer: corrected

 7. Linear and continuous epitopes are the same. In page 8, first paragraph:  do the authors mean to use linear and continuous in the same line?

 Answer: Yes, they are the same and for illustrative purposes the term Epitope lineal is used in the second version.

 8. Discussion, end of paragraph 2:  Do groups A and D share antigenic areas? (what are “affected residues”?).  If so, the explanation is not clear, and not shown in the results.  Maybe an overlay of a representative molecule from each of the two groups showing the areas that are shared in green could be helpful in the results section to illustrate this (and blue and yellow areas in the respective molecules).

 Answer: In our study, we did not find antigenic areas directly related to cross-reactivity between group A and D proteins. However, in the study carried out by Hoffman, et al. 2005 showed that there are levels of identity between the proteins of these groups and they also include Sol i 1 found in group C. Therefore we use this information to compare the results found.

 9. Discussion, third paragraph:  “double positivity” needs to be explained (69% at 75% is not clear). What is OSR? (spell out).

 Answer: the double possibility refers to possible cross reactivities caused by the recognition of different substances contained in the analyzed insects, for example; In the particular case of some bees and wasps, they present double cross-reactivity given by the carbohydrate cross-reaction determinants (CCD) and by phospholipases, as they are different substances but both are recognized in insects, they are called “double positivity”. The percentages show that 69 to 75% of the tests performed for bee venom and yellow jacket venom give double positivity due to the presence of carbohydrate cross-reaction determinants (CCD) and phospholipases, which confuses the diagnosis allergy.

 OSR: Oilseed rape

 This reference explain the concept of positivity double:

 - Hemmer, Wolfgang, et al. Antibody Binding to Venom Carbohydrates Is a Frequent Cause for Double Positivity to Honeybee and Yellow Jacket Venom in Patients with Stinging-Insect Allergy. pp. 1045–52, doi:10.1067/mai.2001.120013.

 10. The discussion is hard to follow, maybe in part because it is not clear from the results if cross-reactivity is expected or not among different species. It might be interesting to have a table in the results section showing the 4 groups with their proteins (in first row and first column) and indicating if clinical cross-reactivity has been observed (also CCD presence or absence if it applies), next to expected cross-reactivities from the results.

 Answer: thanks for the suggestion, we will take it into account to carry out a review of the results, discussion in order to clarify the central ideas and make the information clearer for the reader. We make the suggested table for clarity of information.

**Minor comments:**

 1. English grammar needs revision. For example, in Summary: "phospholipase allergens", "18 amino acid sequences", "share amino acid sequence identity".

 Answer: English grammar will be reviewed.

 2. Some terms need an explanation for readers to understand (for example: “empty spaces”, “length of branches”, “nodes” in the first paragraph, page 4).

 Answer:

 - The empty spaces are areas of different sequences where there is no relationship between their amino acids.

 - The branch length or branch distance refers to the relationship between the allergens exposed in the tree. The longer the branch length, the less related there is between the allergens and the shorter the greater the relationship.

 - Nodes is a synonym for groups or clades that make up the phylogenetic tree.

 3. Page 3, first column, paragraph 2, line 14: "sensitization to this allergen". Must be completed (allergen source?).

 Answer: "sensitization to this allergen" is corrected by "sensitization to hymenoptera allergens" because the central idea revolves around the diversity of allergens of this order.

 4. Page 3, second column, third paragraph, line 10: the sentence with “areas” is vague. Does "area" mean epitopes or something else? Line 11: "Several studies have been carried out ..."

  Answer: The term was corrected for conserved regions that refer to epitopes (these are the regions causing sensitization). What is mentioned on line 11 is support to show that other valid studies have been done.

 5. Page 5, first paragraph: P. Paulista. Names of other species should also be italicized throughout the manuscript (ie, Legend to Figure 6).

 Answer: we italicize those names that were missing.

 7. Legends in Figures 3, 4 and 5 need correction (highly conserved sequences identified, conserved in the middle).

  Answer: Conserved between the sequence with high identity

 8. The verb "Consistent" can be removed from the legend to the figure

 Answer: the verb was removed.
